# The synergistic hydration mechanism and environmental safety of multiple solid wastes in red mud-based cementitious materials

**DOI:** 10.1007/s11356-023-27800-w

**Published:** 2023-06-07

**Authors:** Junge Zhu, Hongzhi Yue, Laijun Ma, Zichao Li, Rong Bai

**Affiliations:** College of Materials Science and Engineering, Shandong University of Technology, Zibo, 255000 Shandong China

**Keywords:** Red mud, Steel slag, Blast furnace slag cement, Flue gas desulfurization gypsum, Fly ash, Hydration reaction principle, Alkali substances, Heavy metal elements

## Abstract

Red mud (RM) is a solid waste material with high alkalinity and low cementing activity component. The low activity of RM makes it difficult to prepare high-performance cementitious materials from RM alone. Five groups of RM-based cementitious samples were prepared by adding steel slag (SS), grade 42.5 ordinary Portland cement (OPC), blast furnace slag cement (BFSC), flue gas desulfurization gypsum (FGDG), and fly ash (FA). The effects of different solid waste additives on the hydration mechanisms, mechanical properties, and environmental safety of RM-based cementitious materials were discussed and analyzed. The results showed that the samples prepared from different solid waste materials and RM formed similar hydration products, and the main products were C–S–H, tobermorite, and Ca(OH)_2_. The mechanical properties of the samples met the single flexural strength criterion (≥ 3.0 MPa) for first-grade pavement brick in the *Industry Standard of Building Materials of the People's Republic of China-Concrete Pavement Brick*. The alkali substances in the samples existed stably, and the leaching concentrations of the heavy metals reached class III of the surface water environmental quality standards. The radioactivity level was in the unrestricted range for main building materials and decorative materials. The results manifest that RM-based cementitious materials have the characteristics of environmentally friendly materials and possess the potential to partially or fully replace traditional cement in the development of engineering and construction applications and it provides innovative guidance for combined utilization of multi-solid waste materials and RM resources.

## Introduction

Urbanization and industrialization are two crucial processes that generate large amounts of solid waste. The environmental problems caused by solid wastes have attracted the extensive attention from many scholars and experts. Red mud (RM) is a strongly alkaline class II solid waste resulting from production of alumina. Depending on the processed raw material, 1–1.5 tons of RM can be produced for each ton of alumina produced, representing 35–40% of the total bauxite (Mukiza et al. 2019a). The utilization ratio of RM is reported to be less than 5% (Wang et al. 2021; Zhang et al. 2020a). The main reason for the difficulty in using RM as a resource is its low activity and strong alkalinity. Moreover, the storage of RM occupies land and further pollutes the land and groundwater with heavy metal elements due to rainwater erosion. Therefore, it is urgent to develop an effective method for utilization of industrial solid waste resources.

Over the past decades, many experts and scholars have attempted to utilize RM. Ti, Fe, Al, and rare earth elements recovered from RM were used as adsorptive materials to remove sewage pollutants in the environment (Klauber et al. 2011). These substances also acted as catalysts and neutralizing agents (Tuazon and Corder 2008; Snars and Gilkes 2009). Although utilization of RM has achieved remarkable results in the above fields, the consumption of RM by such applications is still low due to technical or economic limitations (Zhang et al. 2020b).

RM contains certain amounts of Al and Si oxides and hydroxides, which provides the basis for formation of cementitious materials (Zhang et al. 2011). Therefore, the construction and civil engineering fields could realize the massive utilization of RM. The preparation of cementitious materials with RM and other solid wastes has become an active research area. Cementitious materials prepared from RM, aggregate, flue gas desulfurization fly ash (FGDFA), and cement showed good mechanical properties and durable water resistance (Mukiza et al. 2019b). The composite cementitious material prepared from RM, steel slag (SS), and desulfurized gypsum (DG) significantly increased the overall hydration of the composite material and made the microstructure of the sample compact (Hao et al. 2022). Engineering cement-based composites prepared with RM instead of FA exhibited good tensile strain capacity (Kan et al. 2022). The composite cementitious material prepared from RM and phosphogypsum promoted hydration and changed the pore structures of hydration products, thereby helping to establish a low permeability and high mechanical strength matrix (Huang et al. 2022).

RM-based multi-cementitious materials are kind of alkali-activated cementitious materials; BFSC and metakaolin are two typical raw materials for the preparation of C-(A)-S-H and N-A-S-H cementitious (Li et al. 2023); water glass solution as an exciter combined with coal gangue can produce a compressive strength of 20 MPa cementitious material (Liu 2022); high carbon FA and low alkaline Na_2_CO_3_ solution can also prepare better compressive strength of cementitious materials at cured 28 days (Zhu et al. 2023); these studies are inspiring for further study on RM-based cementitious materials. In addition, the environmental safety of RM-based cementitious materials is an important factor that cannot be ignored. When RM, cement, calcium carbide slag (CCS), and phosphogypsum were combined to produce cementing materials, the permeability coefficient was low, the corrosion was poor under the condition of ideal strength, and Cd^2+^ was not detected in the leaching solution (Cao et al. 2022). After curing for 28 days, the cementing materials prepared from RM, FA, and cement showed Hg, Cr, As, and Pb contents lower than the standard limit for drinking water.

Although synergistic preparation of cementing materials from RM and various solid wastes has been studied from many perspectives, the mechanisms for synergistic hydration between RM and many solid wastes have not been clearly studied, and good coordination between mechanical properties and environmental safety performance with RM-based cementing samples is rarely observed. Therefore, the synergistic hydration mechanism of RM and multiple solid wastes needs further research, and its environmental safety must be considered along with the strength of cementitious materials. RM-based cementitious materials to partially replace traditional cement as the development goal, without producing secondary pollution with a lower carbon footprint advantage to the application and promotion of cementitious materials, give important significance (Zhang et al. 2023).

The main purpose of this work is to take RM as the main raw material and add SS, OPC, BFSC, FA, and FGDG to prepare cementitious materials, respectively. During the co-hydration of RM multi-solid materials, CaO is converted to Ca(OH)_2_ that reacts with silica-alumina components to generate C-S-H and C-A-H gels by volcanic ash reaction, and the gels absorb a large amount of water to improve the denseness of cementitious materials (Song 2022). SO_3_ reacts with calcium components to generate CaSO_4_, which reacts with C-S-H and C-A-H reacts to produce calcareous alumina (AFt) that improves the mechanical properties of the cementitious material (Hou 2019). The cooperative hydration mechanism, mechanical properties, and environmental safety of RM and other solid wastes were analyzed with an X-ray diffractometer, a TA-Q600 synchronous thermal analysis instrument, a Quanta 250-field emission environmental scanning electron microscope, a Nicolet 5700 Fourier transform infrared spectrometer, an HD-2001 low-background multichannel gamma spectrometer, and an Agilent 7500CE inductively coupled plasma–mass spectrometer (ICP-MS7500CE). This paper provides a theoretical basis and a new research perspective for preparation of cementitious materials via synergistic hydration of RM and other solid wastes.

## Materials and methods

### Raw material characterization

RM, SS, OPC, FGDG, FA, BFSC, lime, anti-carbonation coating for concrete (CPC(L)), and sodium silicate solution were used in the present study. The RM came from the Shandong Branch of Aluminum Corporation of China. SS was supplied by Laiwu Iron and Steel Group Co., LTD. OPC and BFSC were provided by Shandong Mountain Aluminum Cement Co., LTD. FA and FGDG were provided by the Qilu Petrochemical Electric Heating Plant of China. The lime was analytically pure from the laboratory. CPC(L), a high-performance polymer additive, was purchased from Zhengzhou Wilis New Building Materials Co., LTD. The sodium silicate solution was made in the laboratory, and the concentration was 56.25% (the mass ratio of sodium silicate analysis of pure: deionized water = 1.6875:3).

**Fig. 1:**
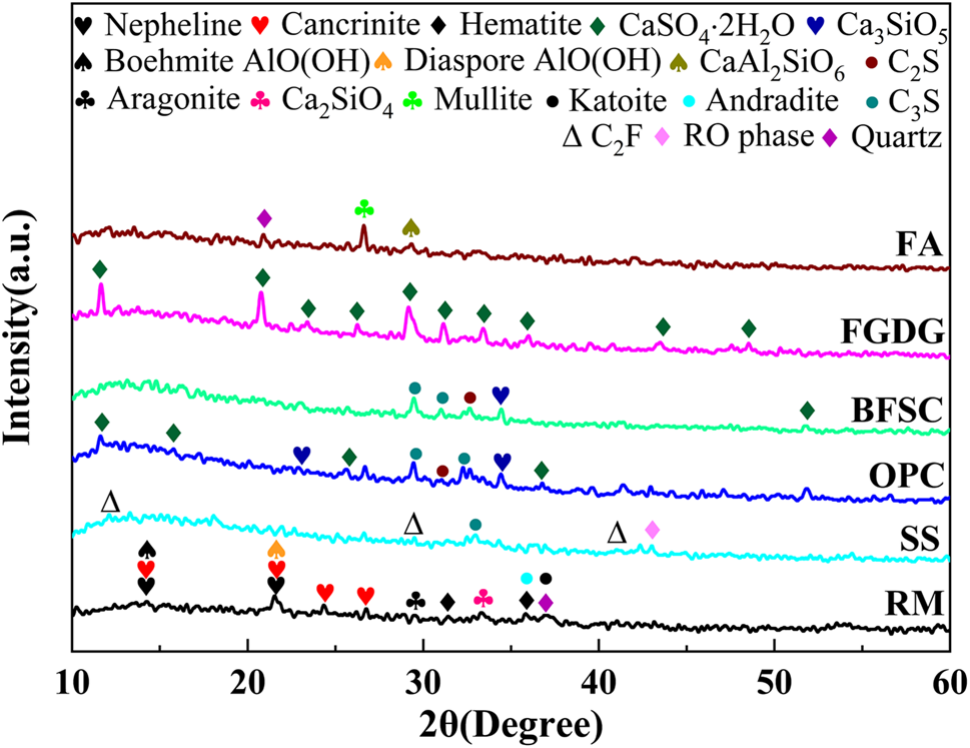
Mineralogical phases of raw materials

The chemical compositions of the raw materials were quantitatively analyzed by X-ray fluorescence (XRF) spectroscopy using a ZSX100e X-ray fluorescence spectrometer made by Nippon Corporation Science, Japan, and the results are presented in Table 1. The mineralogical phases of the raw materials were analyzed with a Bruker AXS D8 ADVANCE X-ray diffractometer, and the results are shown in Fig. 1.

**Table 1:** Chemical composition of raw materials wt%

Constituents	Al_2_O_3_	SiO_2_	Fe_2_O_3_	TiO_2_	Na_2_O	MgO	CaO	MnO	SO_3_	K_2_O
RM	12.2	11.1	30.9	2.65	6.27	0.832	1.75	0.149	0.593	0.144
SS	6.01	18.9	19.4	1.01	0.413	5.39	34.5	3.10	0.298	0.385
OPC	8.46	23.3	3.36	0.632	0.409	3.94	54.1	0.196	4.15	1.00
BFSC	7.91	17.4	1.84	0.468	0.811	3.59	46.8	0.117	3.33	1.00
FGDG	0.894	1.65	7.22	1.49	0.138	0.926	36.9	0.329	40.9	0.0281
FA	14.8	23.6	8.26	0.688	0.367	1.85	14.9	0.861	2.31	0.592

Table 1 shows that the SiO_2_ and Al_2_O_3_ contents of the RM were only 11.1% and 12.2%, respectively. SS, OPC, BFSC, and FGDG are rich in CaO, and FGDG also contains more SO_3_. The SiO_2_ contents of OPC and FA were more than 20%. Mineralogically, the main phases in RM were cancrinite ((Na, Ca, K)_7-8_[(Si, Al)_12_O_24_](CO_3_, OH)_2_‧2–3H_2_O), boehmite AlO(OH), and diaspore AlO(OH). C_2_S, C_3_S, and CaSO_4_‧2H_2_O were present in OPC and BFSC, while Ca_3_SiO_5_ was also in OPC. FGDG contained mostly CaSO_4_‧2H_2_O. The main phases in SS were the C_2_F, C_3_S, and RO phases (complete solids composed of MgO, FeO, MnO, and f-CaO). The main phases in FA were quartz (SiO_2_), mullite (3Al_2_O_3_‧2SiO_2_), and CaAl_2_SiO_6_.

### Experimental procedures

Different proportions of raw materials were used, as shown in Table 2. The flexural strengths were tested to explore the synergistic mechanism for hydration of RM made with different solid waste additives.

**Table 2:** Sample formula table

Mix name	Proportion (dry wt%)				
A	RM	SS	Lime	----	Agglomerant
	60	30	10	----	4.5
E	RM	OPC	----	----	Agglomerant
	60	40	----	----	4.5
F	RM	BFSC	----	CPC(L)	Agglomerant
	60	34	----	6	4.5
G	RM	FA	Lime	FGDG	Agglomerant
	60	24.5	10.5	5	4.5
H	RM	FA	Lime	----	Agglomerant
	60	30	10	----	4.5

----: Blank contrast; agglomerant: sodium silicate solution.

**Fig. 2:**
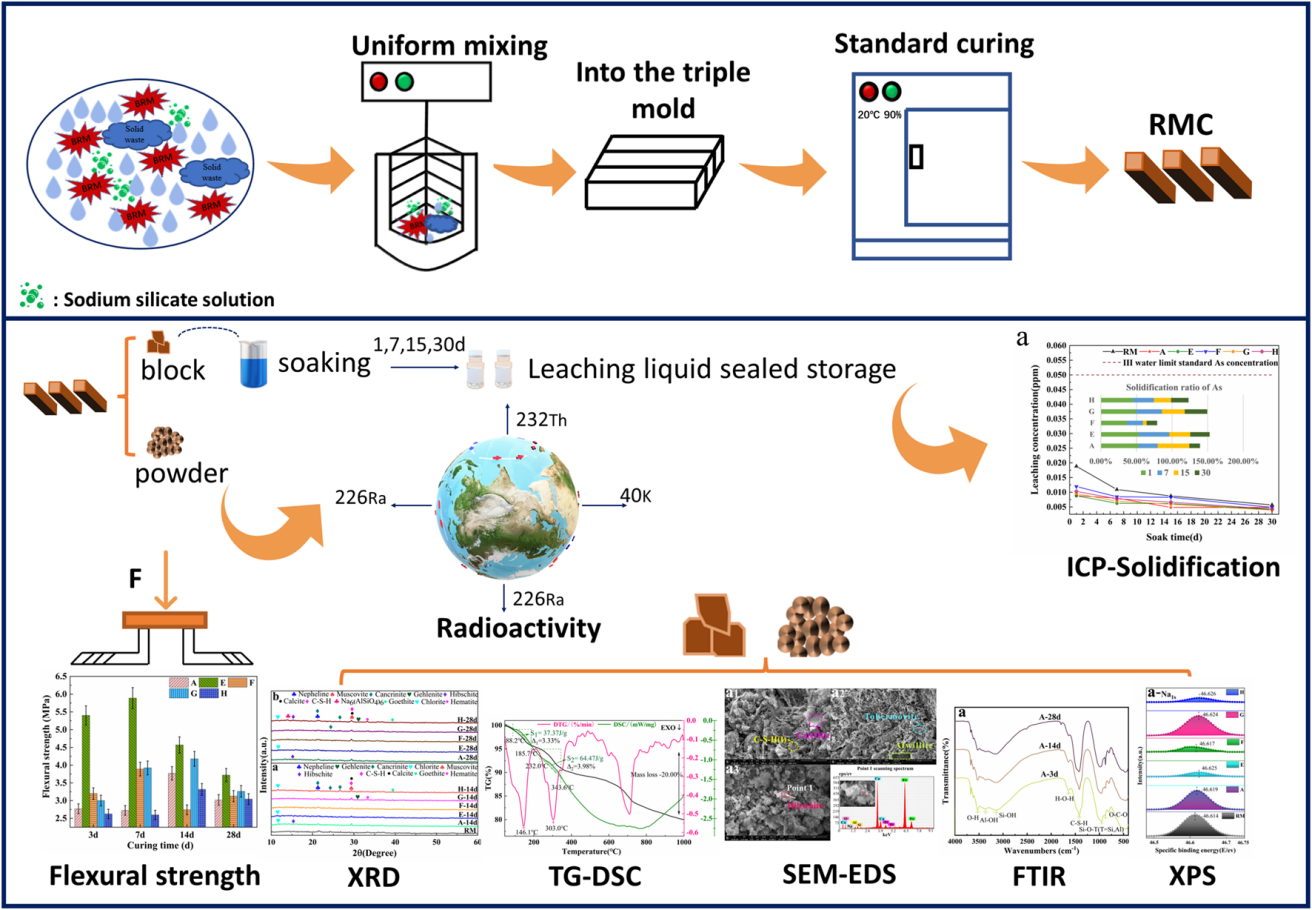
Flow chart of sample preparation and testing

The raw materials were mixed after drying and ball milling according to Table 2, and the water-cement ratio used to prepare the mixed slurry was 0.3–0.5; the slurry samples were put into standard sand molds (40 mm × 40 mm × 160 mm) and named A, E, F, G, and H. Based on *General concrete mechanical properties test method standard* (GB/T 50081-2002 2002), the samples were demolded after curing for 24 h, and then cured for 3 days, 7 days, 14 days, and 28 days, which named RMC uniformly. RMC of different ages were tested for flexural strength. In particular, the RMC cured for 28 days were dried and broken. According to *Solid Waste. Leaching toxicity leaching method. Acetic acid buffer solution method* (HJ/T 300-2007 2007), the extraction agent (2#) was selected, and the 28-day RMC were soaked for 1 days, 7 days, 15 days, and 30 days. According to the experimental program, RMC aged for different times were selected for drying and grinding, and the powder was sealed and stored for subsequent detection (Fig. 2).

According to the literature research, it is known that the proportion of agglomerate in the cementing material should be less than or equal to 10% (Wang et al. 2019). After several experiments, combined with the water-cement ratio and slurry viscosity to determine the agglomerant should be less than or equal to 5% of the powder; this study determined the amount of agglomerant is 4.5%

### Test conditions

Mechanical properties were tested with a WDW-20 electronic universal testing machine. The mineral facies were analyzed with a ZSX100e X-ray fluorescence spectrometer made by Nippon Corporation Science, and thermal analyses of the hydration reaction were performed with a TA-Q600 synchronous thermal analysis instrument. The morphologies of the hydration products were observed with a Quanta 250-field emission environmental scanning electron microscope. The infrared spectra of the hydration products were observed with a Nicolet 5700 Fourier transform infrared spectrometer. The specific activity of radioactivity was determined by using an HD-2001 low background multichannel gamma spectrometer. The Na_1s_, As_3d_, and Cr_2p_ binding energies were determined with a Thermo Scientific ESCALAB Xi+ X-ray photoelectron spectrometer. The heavy metal elements in the RMC extract were tested with an Agilent 7500CE inductively coupled plasma–mass spectrometer (ICP-MS7500CE).

## Results and discussion

### Mechanical property analysis

**Fig. 3:**
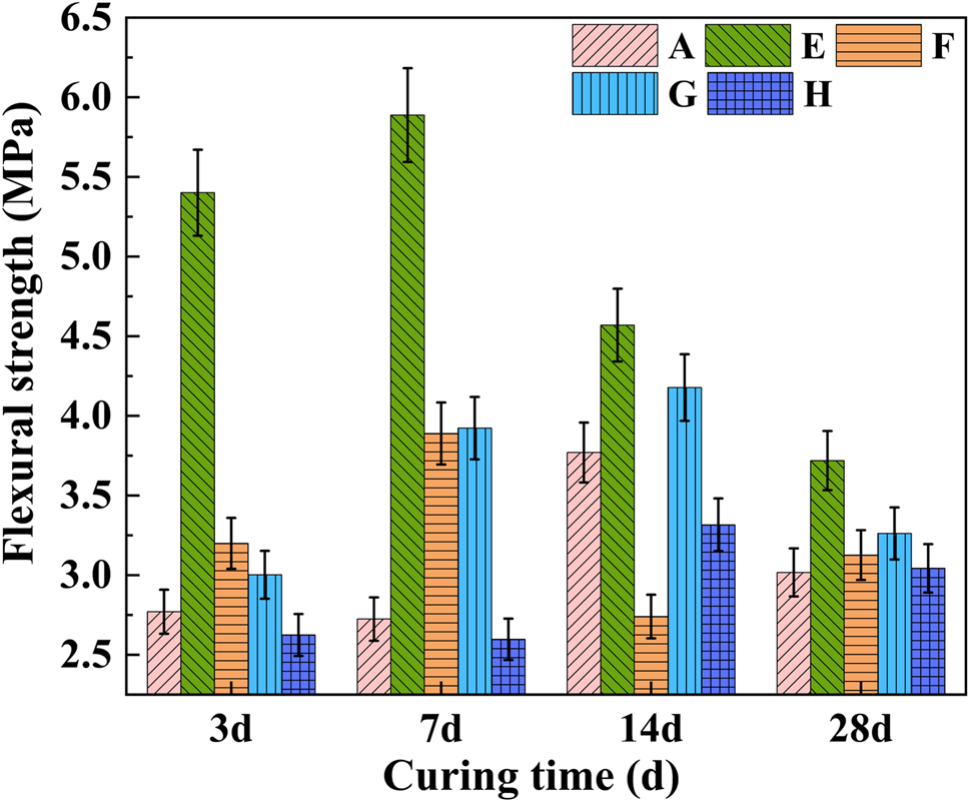
Flexural strength of samples at different curing times

Figure 3 displays the flexural strengths of samples prepared from RM and different solid waste materials with different curing times.

The flexural strength of sample A before 7 days was lower than that after 7 days. That was because the SS in sample A contained C_2_S, which had low hydration activity, resulting in the early low strength of the sample. The flexural strength of sample E in these four groups was the highest because of the calcareous alumina (AFt) generated by the reaction of OPC and RM. Due to the alkaline environment provided by the RM, the calcium oxide in BFSC and the high-performance polymer assistant (CPC) produced C-S-H gel in the early stage of sample strength formation. However, with the extension of the curing time, the combination of CPC and free water molecules in sample F reduced its cohesion. As a result, the samples appeared loose at 28 days. Therefore, the flexural strength of sample F was relatively low. The difference between samples G and H laid in whether FGDG was added. The flexural strength of sample G was greater than that of sample H because there was more SO_3_ in the FGDG of sample G, which was one of the main components of AFt. According to the building materials industry standard of the *Concrete pavement blocks* (JC/T 446-2000 1991), the 28-day flexural strength of samples A, E, F, G, and H met the single flexural strength requirement for first-grade pavement brick (≥ 3.0 MPa).

### XRD analysis

**Fig. 4:**
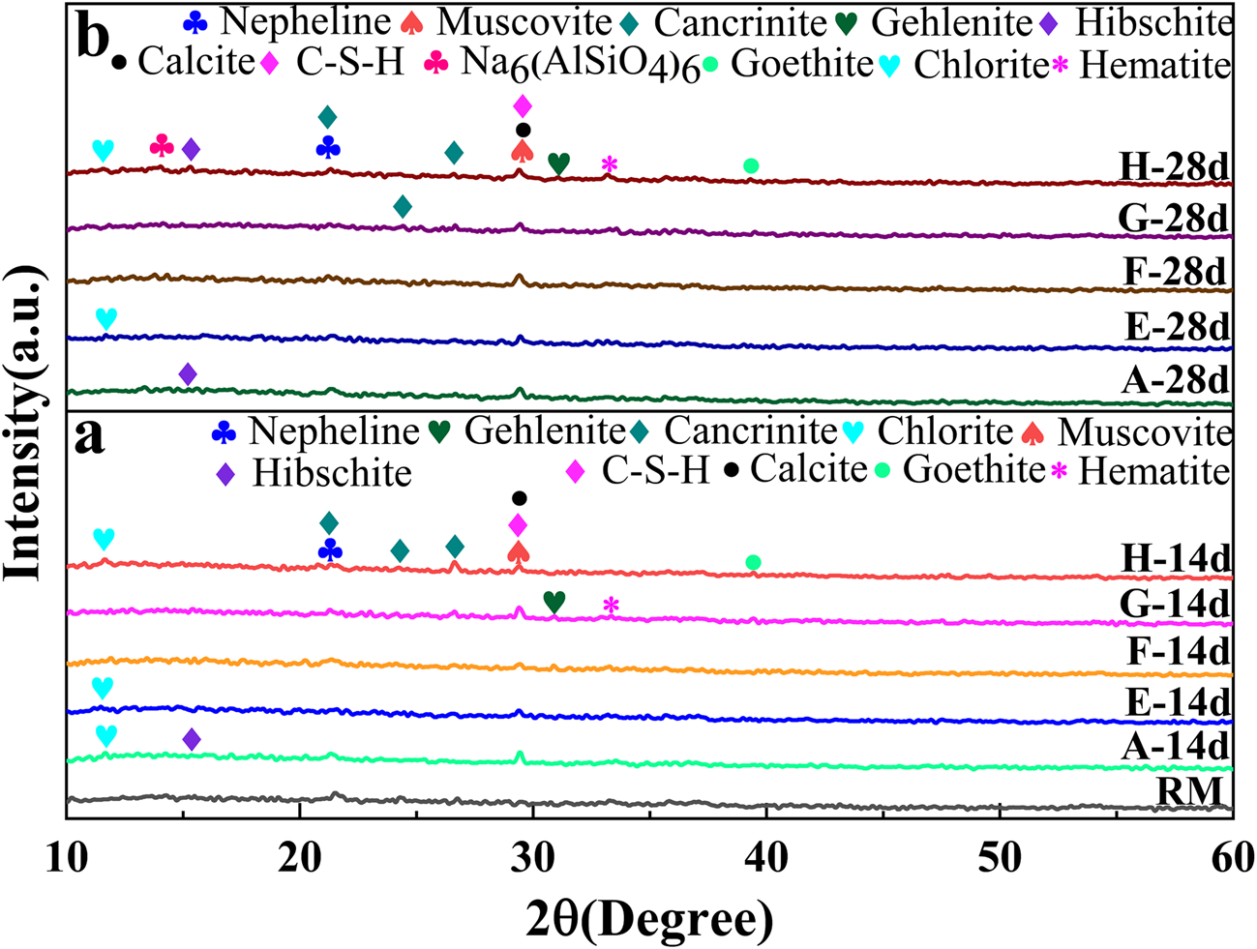
XRD patterns of the samples. **a** XRD patterns of samples at 14 days; **b** XRD patterns of samples at 28 days

Figure 4 presents the phase analyses of raw RM and five group samples aged for 14 days and 28 days. Generally, the five group samples produced C-S-H gel at 29°, which revealed that the combination of different solid wastes and RM supported gelling hydration reactions. The main phases produced by these five groups of samples were nepheline (NaAlSiO_4_), muscovite (KAl_2_(AlSi_3_O_10_)(OH)_2_), cancrinite (((Na, Ca, K)_7-8_[(Si, Al)_12_O_24_](CO_3_, OH)_2_‧2–3H_2_O)), gehlenite (2CaO‧Al_2_O_3_‧SiO_2_), hibschite (Ca_3_(Fe, Al)_2_[SiO_4_]_2_(OH)_2_), calcite (CaCO_3_), Na_6_(AlSiO_4_)_6_, goethite (FeO(OH)), chlorite (Fe_3_[(Si/Al)_4_O_10_](OH)_2_‧Fe_3_(OH)_6_), and hematite (Fe_2_O_3_). The XRD spectra of the samples at 14 days and 28 days were slightly different. Sample A prepared with RM, lime, and SS as the main materials produced chlorite (Fe_3_[(Si/Al)_4_O_10_](OH)_2_‧Fe_3_(OH)_6_) at 14 days, but it disappeared at 28 days, which indicated that there was not enough Si and Al matrix involved in the hydration reaction to produce chlorite at 28 days.

The main phases of samples prepared from RM and OPC were calcite (CaCO_3_) and muscovite (KAl_2_(AlSi_3_O_10_) (OH)_2_), which are important for strength. Sample F was prepared by mixing RM, BFSC, and CPC(L). Due to the complexity of the reaction, the peaks for sample F at 14 days and 28 days were different, and the hydration product peak of sample F was more obvious at 28 days. The phase changes of samples G and H mainly involved the emergence and disappearance of gehlenite (2CaO‧Al_2_O_3_‧SiO_2_), chlorite (Fe_3_[(Si/Al)_4_O_10_](OH)_2_‧Fe_3_(OH)_6_), hibschite (Ca_3_(Fe, Al)_2_[SiO_4_]_2_(OH)_2_, and Na_6_(AlSiO_4_)_6_.

It is worthwhile to note that RM-based cementitious materials undergo many complex reactions, and the XRD patterns of the samples showed mixed peaks, which only preliminarily determined the hydration reactions of the samples. Further hydration reactions are explained in the phase morphology analysis section.

### SEM-EDS morphology analysis

**Fig. 5:**
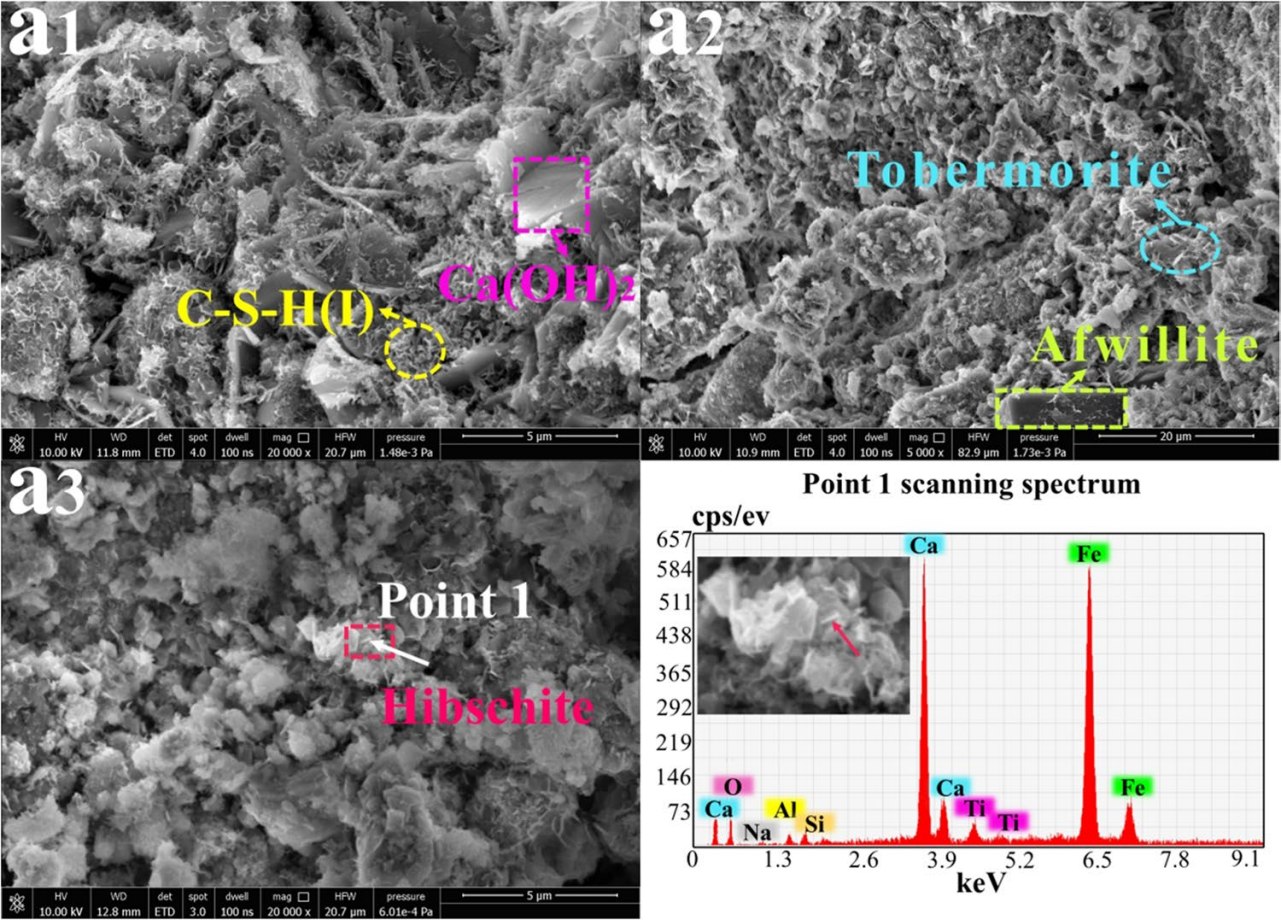
Morphology analysis of sample A at 3 days, 14 days, and 28 days (5000×~ 20000x): **a**_**1**_ A-3 days; **a**_**2**_ A-14 days**; a**_**3**_ A-28 days

**Fig. 6:**
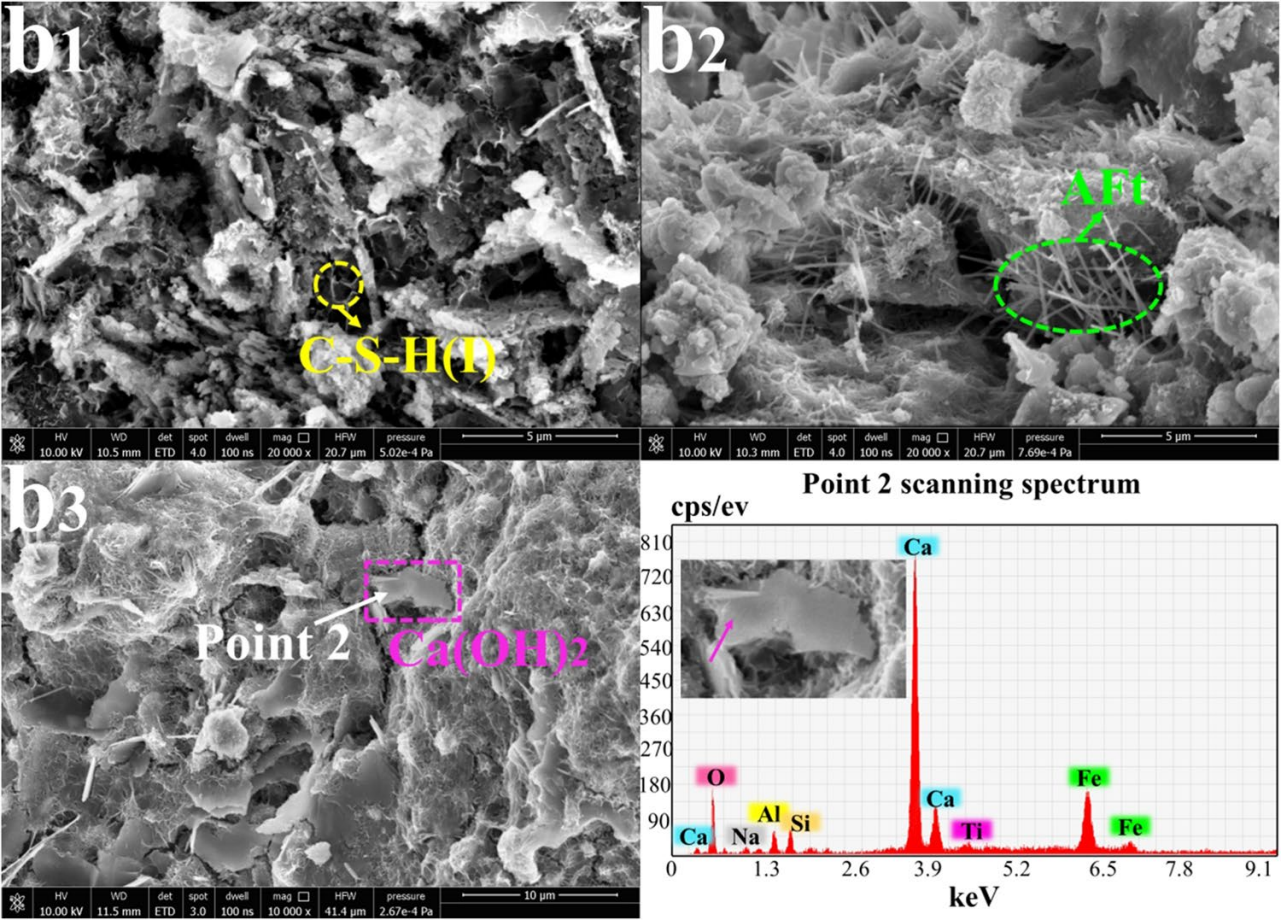
Morphology analysis of sample E at 3 days, 14 days, and 28 days (10000×~ 20000x): **b**_**1**_ E-3 days; **b**_**2**_ E-14 days; **b**_**3**_ E-28 days

**Fig. 7:**
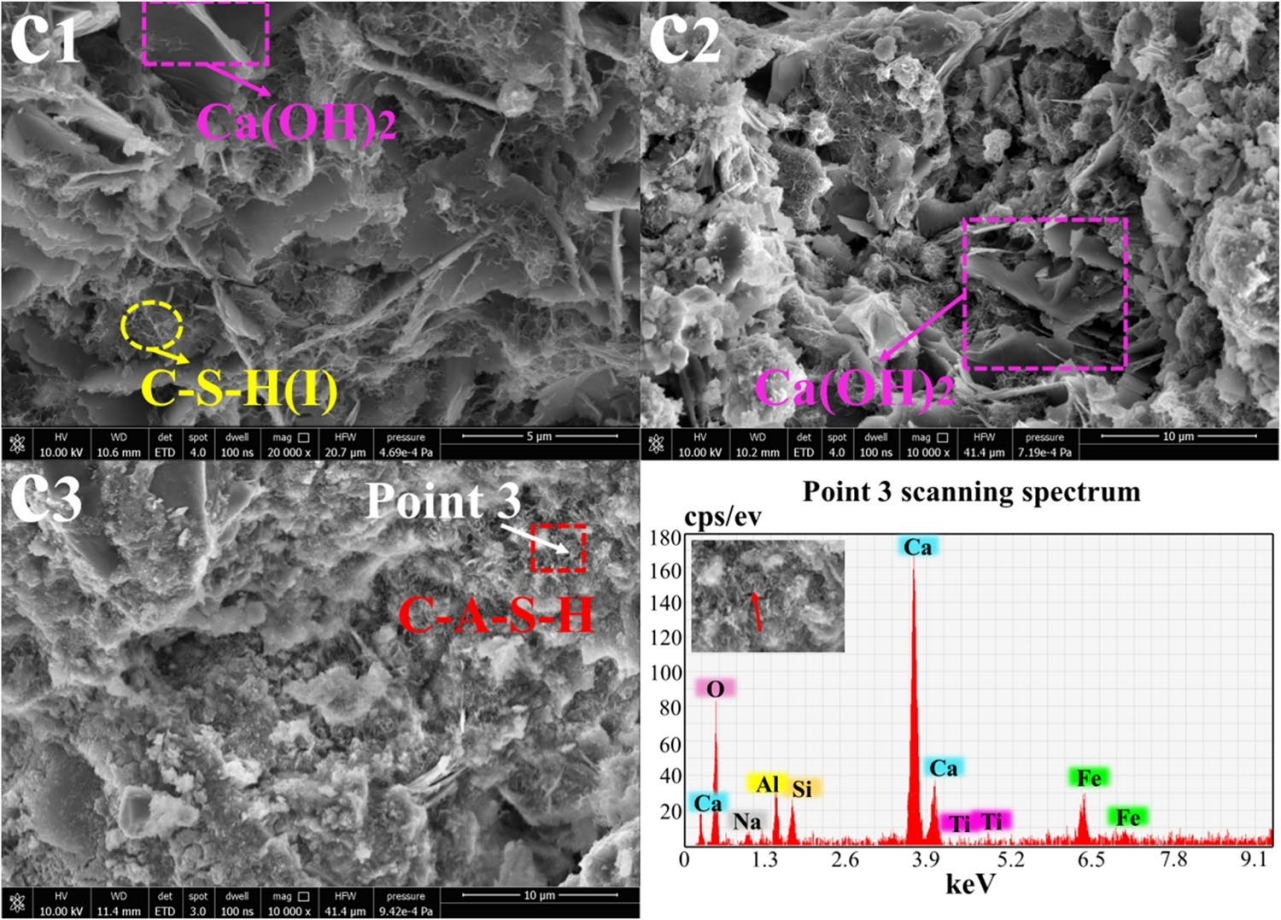
Morphology analysis of sample F at 3 days, 14 days, and 28 days (10000×~ 20000x): **c**_**1**_ F-3 days; **c**_**2**_ F-14 days; **c**_**3**_ F-28 days

**Fig. 8:**
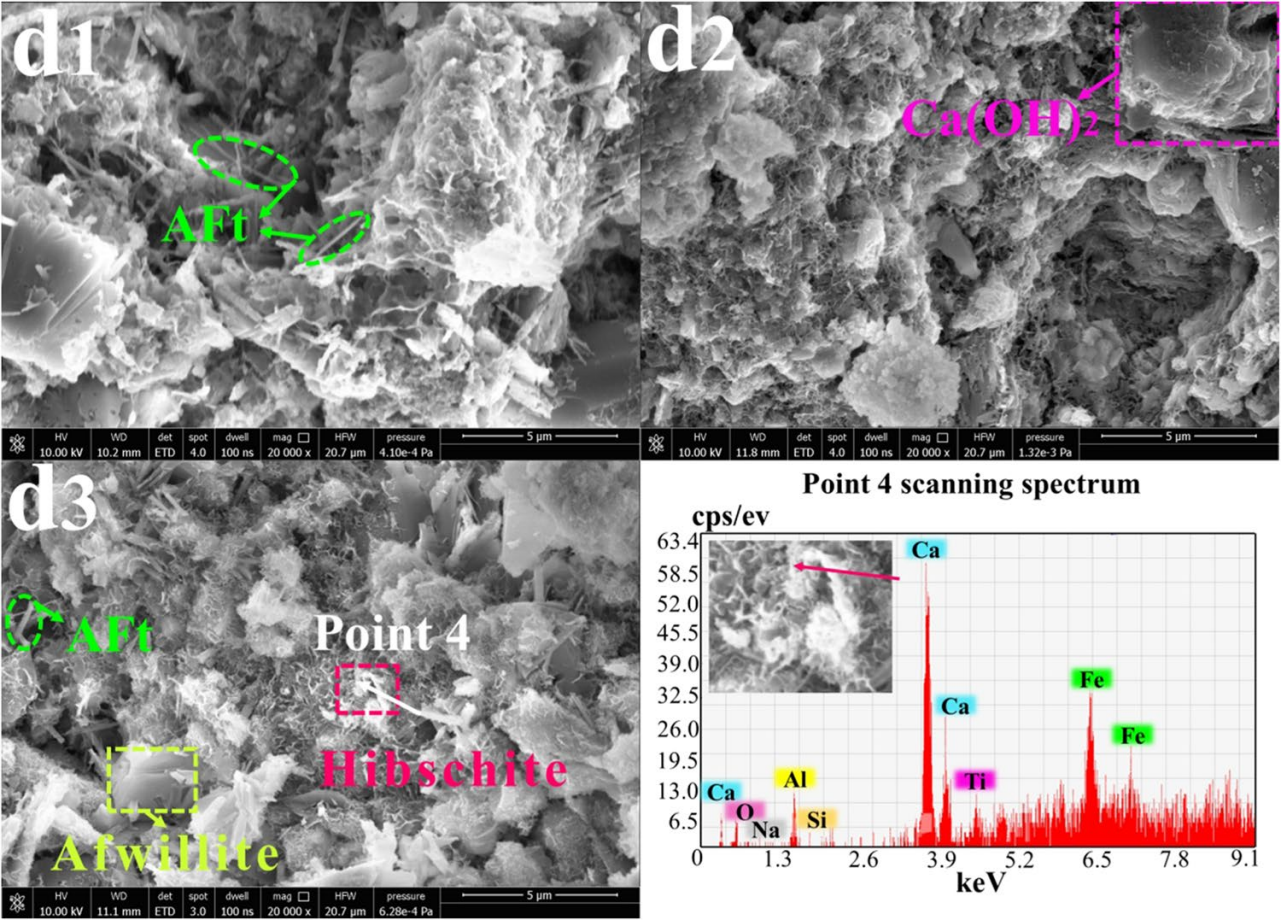
Morphology analysis of sample G at 3 days, 14 days, and 28 days (20000x): **d**_**1**_ G-3 days; **d**_**2**_ G-14 days; **d**_**3**_ G-28 days

**Fig. 9:**
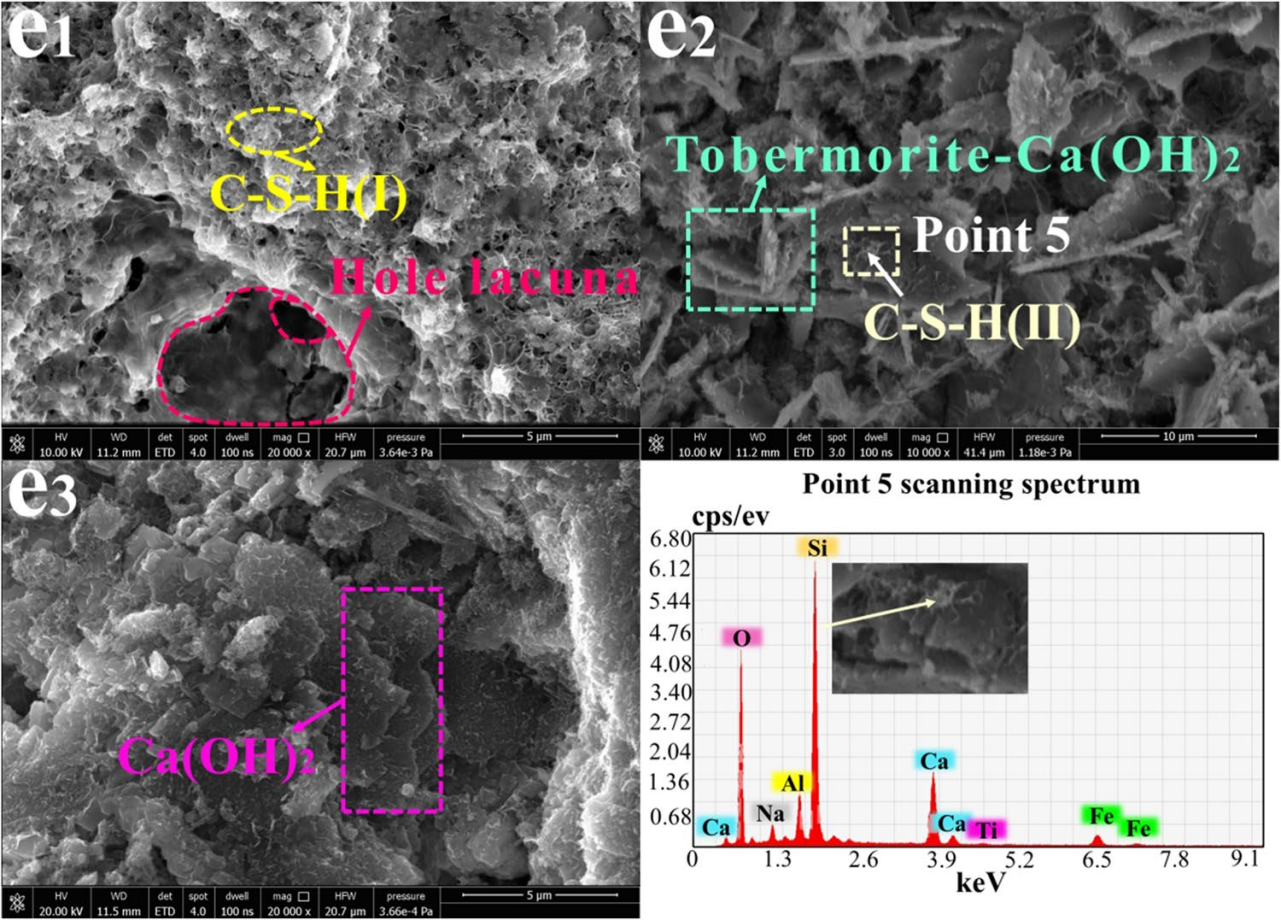
Morphology analysis of sample H at 3 days, 14 days, and 28 days (10000×~ 20000x): **e**_**1**_ H-3 days; **e**_**2**_ H-14 days; **e**_**3**_ H-28 days

Figures 5, 6, 7, 8, and 9 were used to observe the morphological structures of the hydration products in samples A, E, F, G, and H. The morphological structures and element contents of hydration products formed from the combination of different solid wastes with RM materials were different. The hydration products of the above samples were mainly Ca(OH)_2_, tobermorite, C-S-H gel, and a small amount of AFt (Pan et al. 2002; Kong et al. 2018).

Figure 5 shows the microstructure of the sample A prepared with RM, lime, and SS. There were many short curly linear C-S-H crystals, and a few broadly lamellar crystals (Ca(OH)_2_) appeared at 3 days. With extension of the curing age, prismatic crystals (afwillite) and thin lamellae with needle-like interwoven crystals (tobermorite) appeared at 14 days. The formation of afwillite was due to dissolution of Ca(OH)_2_ due to homogenization of Ca- and Si-dominated gels during the hydration reaction. Furthermore, sample A at 28 days produced hibschite (Ca_3_(Fe, Al)_2_[SiO_4_]_2_(OH)_2_), which was polyhedral and was interspersed around the raw material particles not involved in the hydration reaction to improve the denseness of the cementitious structure, but the more hibschite was unfavorable to develop the strength that was consistent with the results of Figs. 3 and 4 (Qiao et al. 2014).

Figure 6 displays the morphologies and structures of samples prepared by mixing RM and OPC and aged for 3 days, 14 days, and 28 days. At 14 days, the sample produced multiple clusters of long linear crystals (AFt), which improved the mechanical properties of the sample, as shown in Fig. 3. The sample at 28 days showed more lamellar crystals (Ca(OH)_2_) (Chen et al. 2019) and finely reticulated C-S-H crystals, which indicated that a large amount of SO_4_^2−^ had been consumed in 14 days (Xu et al. 2021; Wei et al. 2022).

Figure 7 shows the micromorphologies of samples prepared from RM, BFSC, and the polymer additive CPC after aging for 3 days, 14 days, and 28 days. Relatively neatly arranged lamellar crystals (Ca (OH)_2_) and short coiled linear crystals (C-A-S-H) appeared in the sample at 3 days, which indicated that CPC improved the polymerization degrees of the hydration products. With the longer curing time, CPC, RM, and BFSC had reacted fully, and CPC had reacted with the OH^−^ provided by Na_2_O in the RM, which increased the flexibility of the sample. The sample showed a trend of decreasing strength, which was related to the hydrophilicities of the polymer additives.

Figure 8 illustrates the microstructure of sample G, which was prepared from RM, lime, FA, and FGDG and aged for 3 days, 14 days, and 28 days, respectively. Due to the high content of SO_3_ in the FGDG, significant AFt was produced in the samples in the early stages of hydration. With extension of the curing period, more small curly crystals of C-S-H and layered Ca(OH)_2_ gradually appeared. Because the RM contained more Fe_2_O_3_, it remained stable during the hydration reaction and generated hibschite (Ca_3_(Fe, Al)_2_[SiO_4_]_2_(OH)_2_). Additionally, C-S-H grew to become AFt, and Ca(OH)_2_ grew to form afwillite.

Figure 9 shows the microstructures of sample H, which was prepared from RM, lime, and FA, at 3 days, 14 days, and 28 days. At the early stages of hydration, a large amount of C-S-H gel was produced in the sample and was evenly distributed. However, the reaction of lime and water formed a clear hole. From 14 to 28 days, more lamellar crystals appeared in the sample and gradually became increasingly integral, which also implied the adequacy of the sample hydration reaction. Because of the large amount of SiO_2_ in FA, the Si-rich hydration product C-S-H (II) was formed in 14 days, and a tobermorite-Ca(OH)_2_ solid solution appeared.

The processes for hydration of samples prepared from RM and different solid waste materials were similar, and the reactions were as follows (Li et al. 2020a):

1 $$\mathrm{Ca}\mathrm{O}+{\mathrm{H}}_2\mathrm{O}\to \mathrm{Ca}{\left(\mathrm{OH}\right)}_2$$

2 $${\mathrm{Ca}}_2{\mathrm{SiO}}_4+{\mathrm{H}}_2\mathrm{O}\to \mathrm{C}-\mathrm{S}-\mathrm{H}+\mathrm{Ca}{\left(\mathrm{OH}\right)}_2$$

3 $$\mathrm{Ca}{\left(\mathrm{OH}\right)}_2+6{\mathrm{H}}_2\mathrm{O}+6{\mathrm{H}}_2\mathrm{O}+{\mathrm{S}\mathrm{i}\mathrm{O}}_2\to {\mathrm{C}\mathrm{a}}_5{\mathrm{S}\mathrm{i}}_6{\mathrm{O}}_{16}\left(\mathrm{OH}\right)\cdot 4{\mathrm{H}}_2\mathrm{O}\left({\mathrm{C}}_5{\mathrm{S}}_6{\mathrm{H}}_5:\mathrm{Tobermorite}\right)$$

4 $$3\mathrm{CaO}\cdot {\mathrm{Al}}_2{\mathrm{O}}_3+{\mathrm{SiO}}_2+{\mathrm{H}}_2\mathrm{O}\to \mathrm{C}-\mathrm{A}-\mathrm{S}-\mathrm{H}$$

5 $$3\mathrm{CaO}\cdot {\mathrm{Al}}_2{\mathrm{O}}_3\cdot 6{\mathrm{H}}_2\mathrm{O}+3\left({\mathrm{CaSO}}_4\cdot 2{\mathrm{H}}_2\mathrm{O}\right)+6{\mathrm{H}}_2\mathrm{O}\to \mathrm{Aft}$$

**Fig. 10:**
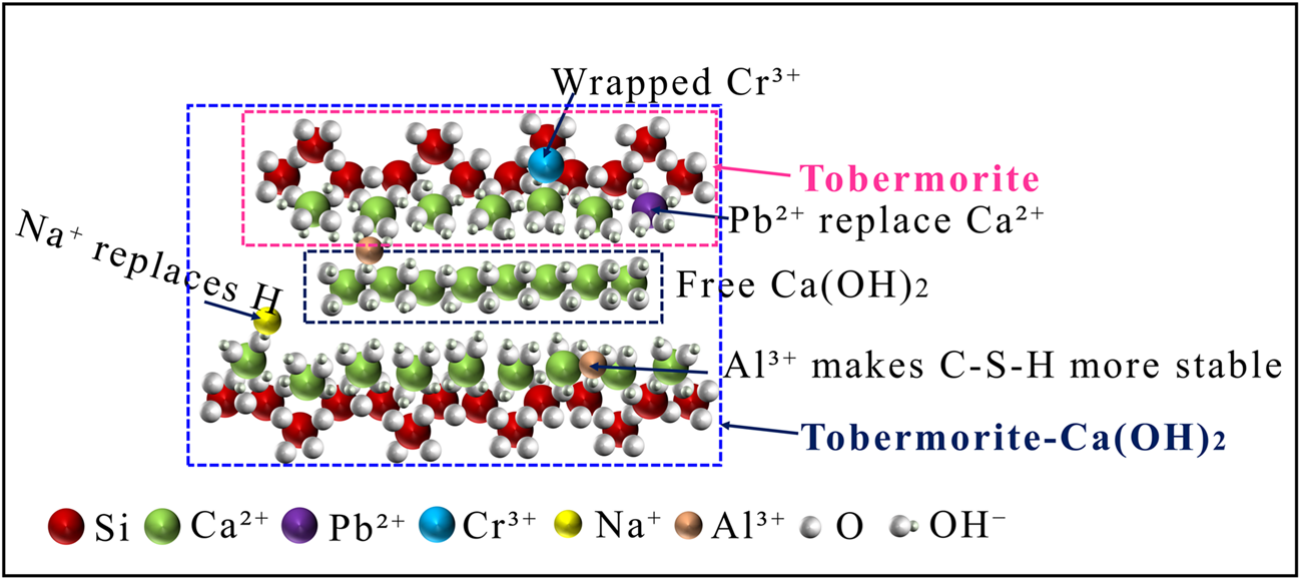
Hydration product formation mechanism

Analyses of the microstructures of the samples showed that the morphology and structure of the hydrated calcium silicate mineral, C-S-H, had different microstructures due to changes in the state and content distribution of CaO and SiO_2_ during the hydration process, which were divided into Ca-rich C-S-H (I) and Si-rich C-S-H (II) (Zhang et al. 2005). Particularly, tobermorite is also a hydrated C-S-H calcium silicate mineral. With extension of the curing time, calcium silicate hydrate might appear as a “solid-solution,” which showed that the Ca(OH)_2_ was adsorbed on the interlayer structure of tobermorite to form a solid solution of tobermorite-Ca(OH)_2_. The formation mechanism of the hydration products is displayed in Fig. 10. Alkali substances and heavy metal elements were also involved in the hydration reaction, which is described in detail in the analysis of elemental leaching, solidification, and specific binding energy.

### FTIR analysis

**Fig. 11:**
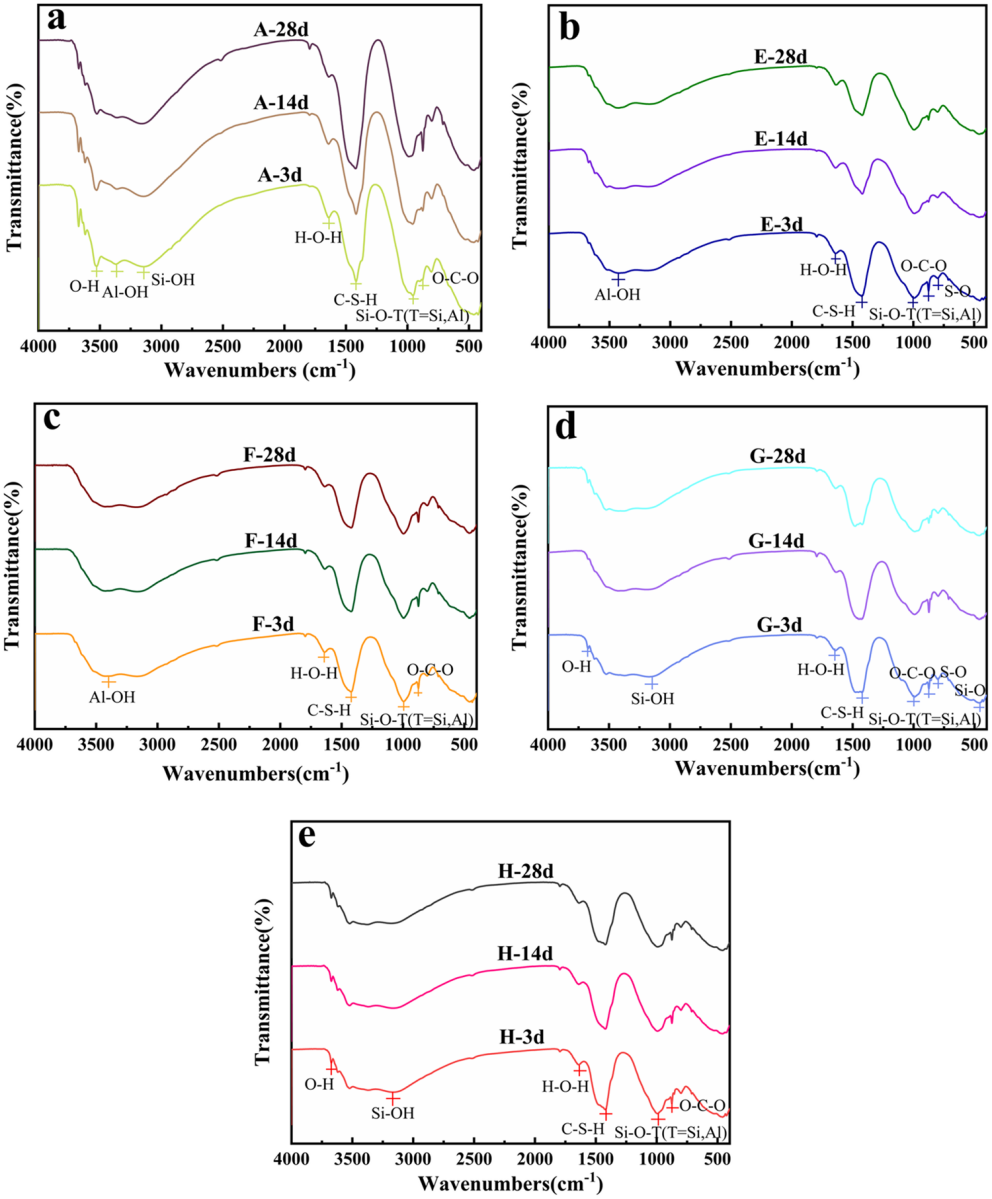
FTIR spectra of different samples at 3, 14, 28 days

Figure 11 shows analysis FTIR spectra in the range 4000–500 cm^−1^ for samples A, E, F, G, and H cured for 3 days, 14 days, and 28 days. The five groups of samples showed similar trends, but there were some differences. Table 3 displays the functional groups indicated by the main bands in the spectra. The absorption band at 3364–3427 cm^−1^ was produced by stretching vibrations of Al–OH in the ettringite octahedron (Liu et al. 2018). The absorption peak at 3527–3675 cm^−1^ originated from O–H stretching vibrations. The band at 3142–3169 cm^−1^ corresponded to the Si–OH stretching vibrations of tobermorite in the hydration products. The band ranging from 1635 to 1796 cm^−1^ corresponded to bending vibrations of the interlayer water molecules. The band at 1416–1422 cm^−1^ was due to the hydration product C-S-H. The band at 954–1002 cm^−1^ corresponded to the antisymmetric stretching vibration of Si–O–T (T = Si, Al), which indicated the simultaneous presence of the hydration products C-S-H and C-A-S-H. The absorption band at 875–876 cm^−1^ represented the bending vibration of O–C–O (CO_3_^2−^) (Huo et al. 2021), which was related to carbonization of the slurry. Especially, the bands at 794–798 cm^−1^ in samples E and G corresponded to S–O bending vibrations, which were related to the gradual participation of SO_4_^2−^ in the hydration reaction (Liu and Na 2011). The band at 455 cm^−1^ in sample G corresponded to a bending vibration of Si–O, which might be related to the decomposition of quartz (Shao et al. 2020; Zhang et al. 2016).

**Table 3:** Wavenumbers corresponding to the functional groups of the samples

Functional group	Wavenumbers/cm−1
O–H	3527–3675
Al–OH	3364–3427
Si–OH	3142–3169
H–O–H	1635–1796
C–S–H	1416–1422
Si–O–T (T= Si, Al)	954–1002
O–C–O	875–876
S–O	794–798
Si–O	455

The transmittance values of bands for functional groups in the same wavenumber range were inversely proportional to the contents of hydration products (Li et al. 2020b). Table 4 shows the transmittance values for the same functional groups in the five groups of samples made with different curing times. The O–H and Al–OH stretching vibrations were related to the formation of ettringite. According to Table 4, the transmittances of O–H and Al–OH of samples E and F were smaller than those of other samples, which indicated that the combinations of RM and OPC and RM and BFSC generated more ettringite. However, there was no obvious Si–OH bending vibration in samples E and F, which indicated that the Si–O tetrahedra in samples E and F were more stable, and the hydration products were more stable. The transmittances of the peaks for Si–O–T (T = Si, Al) in the F samples cured for 3 days, 14 days, and 28 days were 11.11%, 10.05%, and 10.23%, respectively, which were lower than those for other samples with the same curing age, this indicated that C-S-H and C-A-S-H were present in sample E at the same time. These results were consistent with the results of the microstructure morphology analyses.

**Table 4:** Transmittance corresponding to different functional groups of 3, 14, and 28-day samples

%	O–H	Al–OH	Si–OH	H–O–H	C–S–H	Si–O–T	O–C–O	S–O	Si–O
A-3 days	53.97	55.20	53.38	87.31	43.63	33.74	44.7	------	------
E-3 days	------	46.46	------	73.69	15.99	13.99	23.08	39.76	------
F-3 days	------	44.23	------	75.70	23.80	11.11	31.90	------	------
G-3 days	82.12	------	44.54	79.32	23.76	18.34	30.89	41.03	10
H-3 days	84.32	------	42.87	79.20	20.04	14.73	29.01	------	------
A-14 days	46.38	46.90	41.77	78.03	29.86	22.68	33	------	------
E-14 days	------	48.40	------	75.48	41.56	15.24	31.63	36.34	------
F-14 days	------	45.28	------	74.07	18.34	10.05	27.01	------	------
G-14 days	90.70	------	48.91	71.89	10.21	16.17	19.15	40.18	10.14
H-14 days	81.75	------	43.78	76.02	16.76	13.57	24.18	------	------
A-28 days	49.35	46.44	42.24	74.78	11.12	19.70	24.2	------	------
E-28 days	------	44.63	------	76.18	35.15	15.74	31.06	39.42	------
F-28 days	------	45.48	------	71.74	17.81	10.23	25.43	------	------
G-28 days	80.63	------	45.86	72.22	25.52	15.28	27.71	40.03	10.02
H-28 days	80.01	------	45.64	72.80	17.80	14.62	23.78	------	------

------: No obvious vibration band

### TG-DTG-DSC

**Fig. 12:**
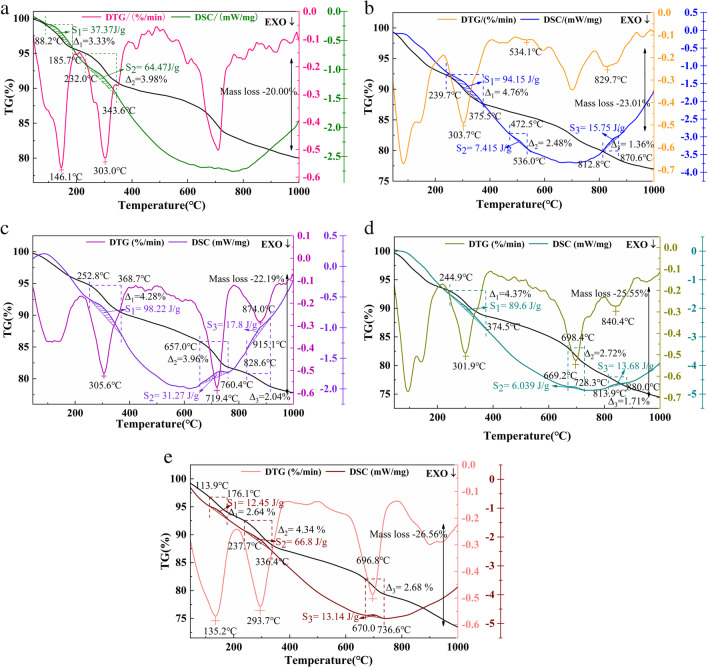
TG-DTG-DSC curves of the samples at 14 days. **a** Sample A; **b** sample E; **c** sample F; **d** sample G; **e** sample H; S: calorific value change; Δ: mass loss

Figure 12 displays the mass losses observed during heating at 45–1000 °C for samples A, E, F, G, and H cured for 14 days. The five groups of samples manifested similar trends for mass change, with mass losses of 20.00%, 23.01%, 22.19%, 25.55%, and 26.56%, respectively. Combined with the XRD and FTIR analyses, this indicated that the continuous weight loss seen in the temperature range 80–400 °C was related to evaporation of free water and removal of bound water from the hydration products C-S-H and AFt. The weight loss observed between 400 and 600 °C was caused by thermal decomposition of the Ca(OH)_2_. The mass loss seen in the temperature range 600–800 °C was related to decomposition of calcite (CaCO_3_). The weight loss occurring in the temperature range of 800–1000 °C was due to removal of residual water molecules from the hydration products.

The mass losses of the samples occurring in different temperature ranges indirectly reflected the corresponding contents of hydration products. In the temperature range 80–400 °C, the mass losses of samples A, E, F, G, and H were 7.31% (3.33% + 3.98%), 4.76%, 4.28%, 4.37%, and 6.98 (2.64% + 4.34%), respectively, indicating that there was more free water present in samples A and H. In the temperature range of 200–400 °C, the calorific values corresponding to removal of water from the hydration products in samples E, F, and G were 94.15 J/g, 98.22 J/g, and 89.6 J/g, respectively, which were larger than those of the other samples. The magnitude of the calorific value might be related to the stability of the substance (Wang et al. 2022), which indicated that the water molecules in samples E, F, and G existed in unstable states. At 400–600 °C, the mass loss of sample E was 2.48%, while those of other samples were nonexistent, which explained why the samples prepared from RM and OPC produced more Ca(OH)_2_ at 14 days. In the temperature range of 600–800 °C, the weight losses of samples F, G, and H were 3.96%, 2.72%, and 2.68%, respectively. At 800–1000 °C, the weight losses of samples E, F, and G were 1.36%, 2.04%, and 1.71%, respectively. This was consistent with the results of the FTIR analyses and the microscopic morphology observation.

### Radionuclide analysis

To further investigate the radioactivity levels of the cementitious materials prepared from OPC-RM and SS-RM, respectively, samples A and E were selected for radioactivity detection. Table 5 shows the radioactivity limits for building materials, and Table 6 demonstrates the radioactivity data for samples A and E. The *I*_γ_ and *I*_Ra_ values of the samples shown in Table 6 were calculated with the following equations.

6 $${\mathrm{I}}_{\gamma }={\mathrm{C}}_{\mathrm{Ra}}/370+{\mathrm{C}}_{\mathrm{Ra}}/260+{\mathrm{C}}_{\mathrm{K}}/4200$$

7 $${I}_{\mathrm{Ra}}={C}_{\mathrm{Ra}}/200$$

**Table 5:** Radioactivity requirements for building materials (GB6566-2010)

Type of materials		Internal exposure index	External exposure index	Applied range
		(Bq/kg)	(Bq/kg)	
Decorative materials	Category A	≤ 1.0	≤ 1.3	Unrestricted range
	Category B	≤ 1.3	≤ 1.9	Cannot be used for class I building interior finishes; can be used for class II civil buildings.
	Category C	---	≤ 2.8	Can only be used for building exterior finishes and outdoors
Main building materials		≤ 1.0	≤ 1.0	Unrestricted range

**Table 6:** Radioactivity assessment of samples

	Specific activity (Bq/kg)			Internal exposure index	External exposure index
	^226^Ra (C_Ra_)	^232^Th (C_Th_)	^40^K(*C*_k_)	*I*_Ra_ (Bq/kg)	*I*_γ_ (Bq/kg)
A	26.56	25.22	63.21	0.1	0.1
E	26.05	14.2	38	0.1	0.2

where *I*_γ_ is the external irradiation index, *I*_Ra_ is the internal irradiation index, *C*_Ra_ is the specific activity of ^226^Ra, *C*_Th_ is the specific activity of ^232^Th, and *C*_K_ is the specific activity of ^40^K.

Tables 5 and 6 show that the internal and external exposure indices of samples A and E were in the unrestricted range for major building materials and decorative materials. RM contains certain natural radioactive substances, which mainly originate from release of ^226^Ra, ^232^Th, and ^40^K (Tian et al. 2015). The specific activities of radionuclides in the minerals formed during hydration of RM and changes in the specific activities of radionuclide caused by adsorption and encapsulation in the cementitious hydration products determined the specific activities of radionuclides in the cementitious material.

Since samples A and E had the same amounts of RM added, the radioactivity studies of these samples mainly considered the capacities of the cementitious hydration products for adsorption and encapsulation of radionuclides. Table 6 shows that the specific activities of ^226^Ra, ^232^Th, and ^40^K in sample E were lower than those in sample A. This indicated that the hydration products of sample E had a denser structure and were more encapsulating for radionuclides. Additionally, the C-S-H in sample E had a large specific surface area and showed strong adsorption and exchange of ions (Lai 2012), which could hold nuclide ions on the surface via physical and chemical adsorptions when it decayed (Lameille et al. 1987). This indicated that cementitious materials prepared from RM and OPC produced more hydration products than cementitious materials prepared by RM and SS.

---: No special requirements

Class II civil buildings: shopping malls, cultural and entertainment venues, bookstores, libraries, gymnasiums, waiting rooms for public transportation, barbershops, etc.

### Analysis of elemental leaching and solidification

The solidification ratio for each element in the samples was calculated according to the following formula:

8 $$m=x/y$$

9 $$k=1-m$$

where *x* is the leaching concentration of each element of the samples (ppm); *y* is the element leaching concentration of RM (ppm); *m* is the dissolution ratio (%); and *k* is the solidification ratio (%).

**Fig. 13:**
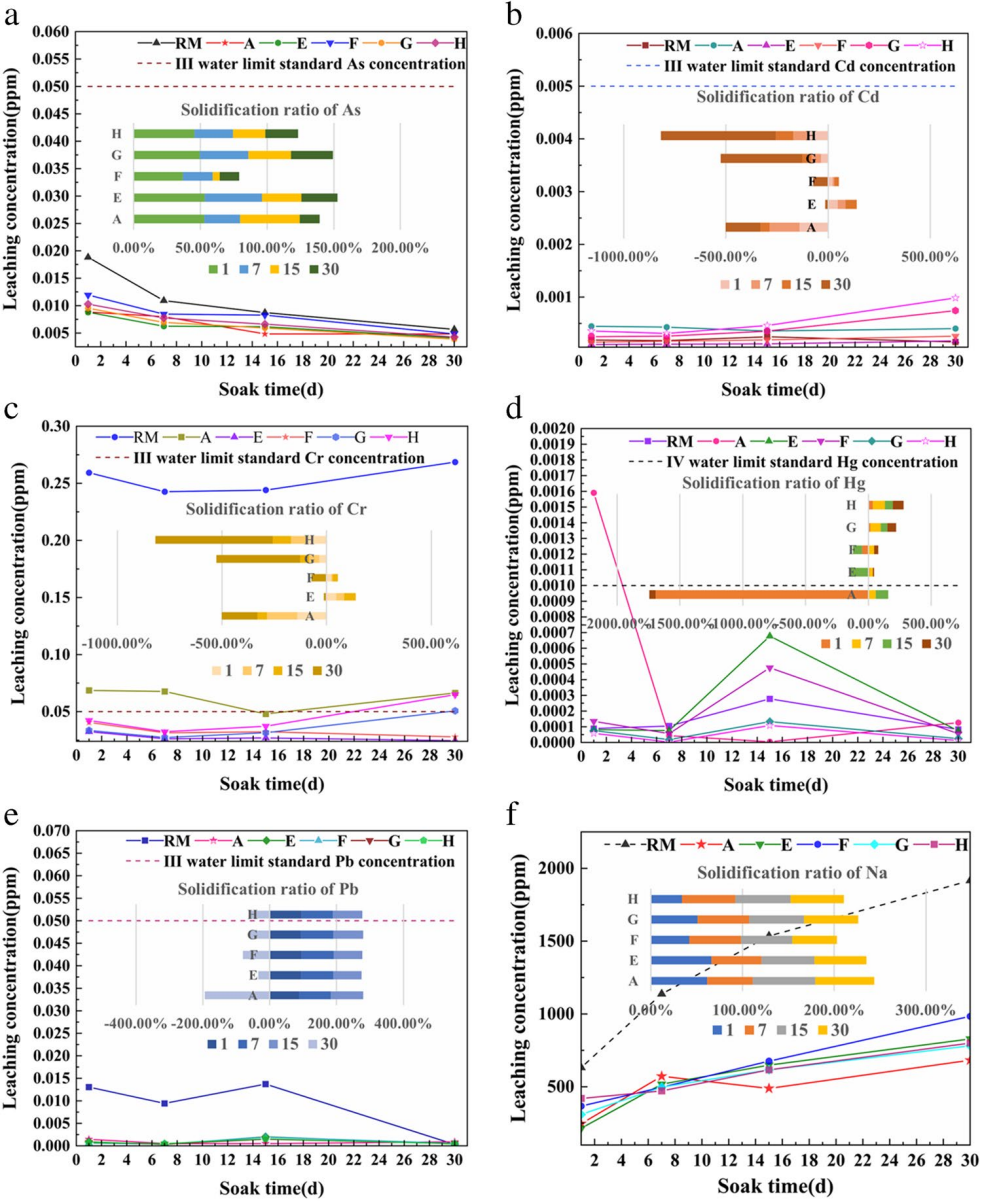
Elemental leaching and solidification ratio of 28 days samples at different soaking times

Figure 13 illustrates the leaching concentrations and solidification conditions for the elements present in the samples after different soaking times. Compared with RM, the leaching concentrations of heavy metal elements in the sample were lower than the class III and class IV standards in the surface water quality standard (Table 7).

**Table 7:** Leaching concentration of heavy metals in samples soaked for 15 days

Leaching concentration	As	Cd	Cr	Hg	Pb
A	0.00484	0.00036	0.0480	0.00000	0.00049
E	0.00616	0.000112	0.027	0.000677	0.00203
F	0.00827	0.000188	0.032	0.000476	0.00196
G	0.00593	0.000354	0.032	0.000133	0.00148
H	0.00662	0.000461	0.037	0.000108	0.00156
III	≤ 0.05	≤ 0.005	≤ 0.05	≤ 0.0001	≤ 0.05
IV	≤ 0.1	≤ 0.005	≤ 0.05	≤ 0.001	≤ 0.05

Figure 13f shows the leaching concentrations of Na in the sample after different soaking times and the solidification ratio of Na relative to the RM. It was found that the leaching concentration of Na in the sample increased with increasing soaking time due to the stable alkaline environment provided by RM as shown in Table 8, the solidification ratios for Na in the five groups of samples were all greater than 55%, which indicated that the five groups of samples had positive effects on fixation of alkali substances.

**Table 8:** The solidification ratio of elements for samples at soaking 15d

Solidification ratio (*k*)	As	Cd	Cr	Hg	Pb	Na
A	44.57%	− 44.52%	− 44.52%	98.38%	96.40%	68.18%
E	29.50%	54.62%	54.62%	− 143.71%	85.24%	57.77%
F	5.24%	24.09%	24.09%	− 71.23%	85.72%	55.95%
G	32.03%	− 43.36%	− 43.36%	52.31%	89.23%	59.84%
H	24.22%	− 86.68%	− 86.68%	61.24%	88.64%	59.88%

Figures 13a, 13b, 13c, 13d, and 13e demonstrate the leaching and solidified states of As, Cd, Cr, Hg, and Pb, which all had low leaching concentrations. The changes seen in the leaching concentrations of elements before and after the 15-day immersion period were different because dissolution of the elements in the early stages of immersion was due to physical diffusion, while the change of element concentration at the later stages was due to dissolution. The sample fixed the elements effectively after 15 days. The leaching concentration of Hg in a sample soaked for 15 days appeared to show an inflection point, which was related to the fact that Hg was initially adsorbed on the surface of the hydration product and then existed freely in the leaching solution. The adsorption of Hg in RM was rapid, and the adsorption kinetic process was best described by Ho’s pseudo-second-order equation (Rubinos David and Teresa 2015). Pb and As could replace Ca in hydration products, while Cr and Cd could be wrapped in the layered structures of the hydration products (Liu et al. 2009). Since the stabilities of elements encapsulated by hydration products were lower than those of the atoms replacing the original elements in the hydration products, the leaching trends for Pb and As gradually came stable, while leaching of Cr and Cd increased slightly in the later stages of immersion, which was consistent with the results shown in Fig. 10. In addition, it has been shown that heavy metals do not move easily in weak acid or reducing conditions, so heavy metals were even less likely to move in the acetate buffer chosen for this study, which guaranteed the environmental safety of the cementitious material (Rubinos David and Teresa 2013).

### Elemental specific binding energy analysis

Based on the solidification levels of the samples, the Na_1s_, As_3d_, and Cr_2p_ specific binding energies of RM, and samples A, E, F, G, and H were selected for analyses. The higher the specific binding energy of an element, the more stable the element is, which is indirectly related to the stabilities of hydration products. The higher the specific binding energy of the element is, the more stable the element is in the hydration product. Due to the high specific surface energies and ion exchange capacities of the hydration products, movements of multiple metal cations could be controlled by physical adsorption, symbiosis, and interlayer chemical substitution. Prior studies have shown that Ca-rich hydration products (C-S-H-I) are more capable of heavy metal solidification and stabilization (Lan et al. 2014).

**Fig. 14:**
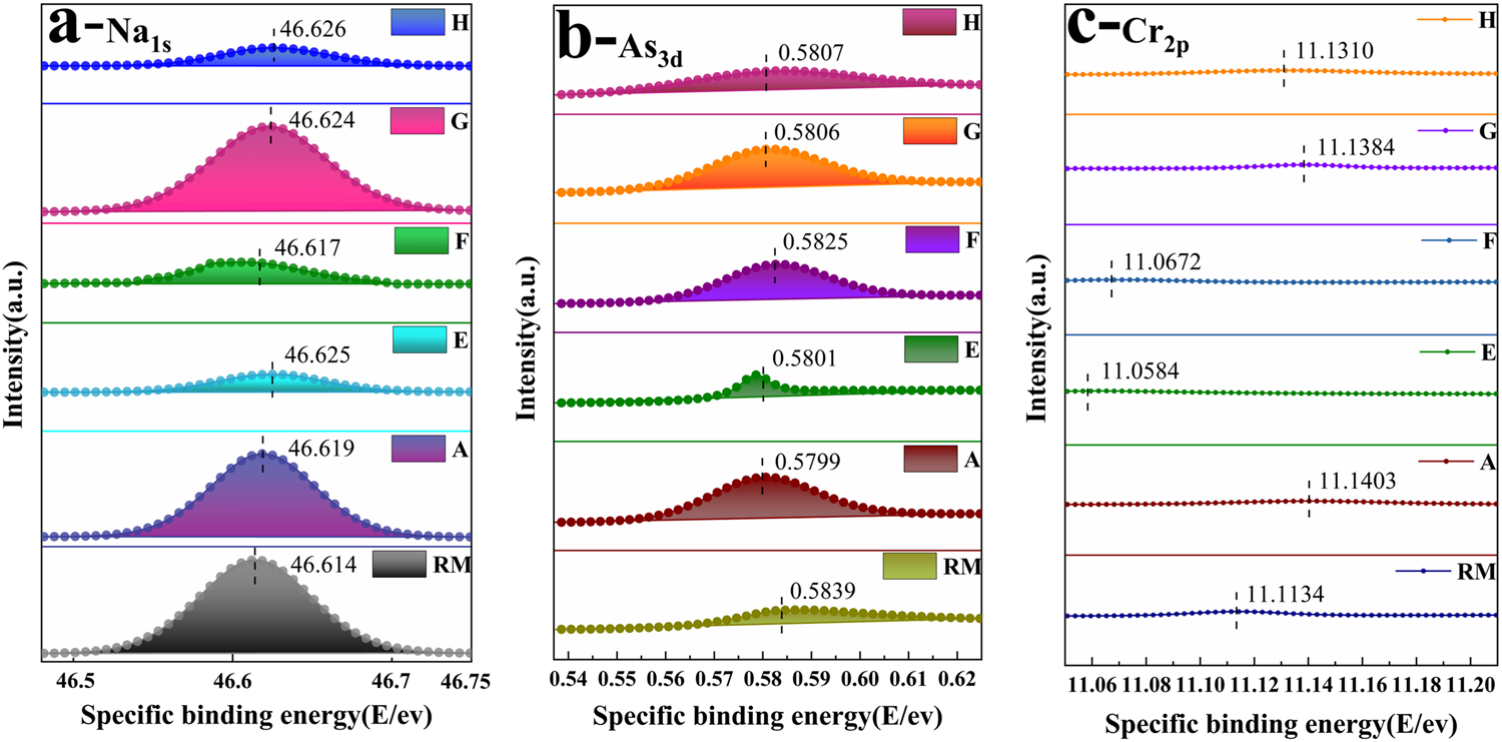
Specific binding energy analysis of Na_1s_, As_3d_, and Cr_2p_ in samples at 14 days

Figure 14a demonstrates the Na_1s_ specific binding energies seen for the RM and other samples. The specific binding energies of Na_1s_ were higher for the five groups of samples compared to the RM, which indicated that the alkali substances were more stable in the cemented samples than in the RM. The specific binding energy ranking was H (46.626E/eV) > E (46.625E/eV) > G (46.624E/eV), which indicated that the additives OPC, FA, and FGDG formed more C-S-H with RM, and Na could replace OH^−^ of Si–OH in C-S-H and participate in the rearrangement and further solidification of hydration products.

Figures 14b and 14c show the As_3d_ and Cr_2p_ specific binding energies for the RM and other samples, respectively. The changes in the specific binding energies of the heavy metals in different samples were not consistent, and it appeared that the binding energies of the samples were either higher or lower than those of RM, which was related to the type of hydration products formed in the samples. The As in the RM was more stable than in the samples, which might be due to the valence diversity of As. It did not adsorb stably in the colloidal samples because the large atomic radius made this difficult. Therefore, it also could not replace metal ions such as Ca^2+^ which in the hydration products with. However, the analysis of As leaching from the 28-day sample showed that the leaching concentration was much lower than the limit for surface water class III, indicating that the sample would not exceed the allowable level for As. Cr has a smaller atomic radius and could squeeze into the interlayer gap and participate in rearrangement of the Si-rich hydration products (C-S-H-II), but it was not effective in rearrangement of the Ca-rich hydration product because the Si-rich hydration products (C-S-H-II) had small spacings between the bridging oxygens in the silica-oxygen tetrahedral chain structure (Taylor 1990); this facilitated the entry of atoms with smaller atomic radius, while the Ca-rich hydration product had a larger chain structure, which could not stabilize atoms with small radius.

Samples E and F were prepared from RM and added OPC or SS, respectively, and the products were Ca-rich hydration products that were not suitable for recombination with Cr, so the specific binding energies of Cr_3p_ in samples E and F were low. Figure 13f shows that the concentrations in the 28-day samples were far below the allowable limits for surface water class III, which provides optimism regarding utilization of these samples.

## Conclusions

In this work, the cementing materials with different activation effects were prepared through addition of steel slag, cement, blast furnace slag cement, fly ash, lime, and flue gas desulfurization to the red mud, respectively. The mechanism for cement hydration and aspects of environmental safety were studied. The results were as follows.

### Mechanical properties

The flexural strength analysis results showed that steel slag, cement, fly ash, and flue gas desulfurization gypsum all showed good activation of red mud, and the samples aged for 14 days and 28 days exhibited single block flexural strengths ≥3.0 MPa.

### Hydration mechanism

Since the solid waste materials added to the red mud had different characteristics; the main mechanism for activation of red mud and production of hydration products involved polymerization and rearrangement of the hydration products to form Ca-rich C-S-H or Si-rich C-S-H.

### Environmental safety

The slow growth in the leaching concentration and the higher specific binding energies for Na in the samples showed that it existed stably in the sample and provided a stable alkaline environment for the hydration reaction. The specific binding energy of As_3d_ in each group of samples was similar. With the analysis of the leaching concentration, it showed that the As element was in a stable state in the sample; however, the adsorption characteristics of hydration products produced by different samples were different. The specific binding energies of Cr_2p_ in samples were higher than 10 E/eV, which indicated that it bound stably with hydration products, and heavy metal elements in the sample remained stable and met the allowable limits of the surface water environmental quality standards for class III and class IV water. Additionally, the radioactive specific activities of ^226^Ra, ^232^Th, and ^40^K in the samples met the unlimited range standard for building main materials and decorative materials.

## Data Availability

Data and materials are contained within the article.
